# Supplementary material for: Adiponectin Deficiency Alters Placenta Function but Does Not Affect Fetal Growth in Mice
Source: Int J Mol Sci. 2022 Apr 29;23(9):4939. doi: 10.3390/ijms23094939 (PMC9101632; doi:10.3390/ijms23094939)
Supplement: Supplementary file 1 [file ijms-23-04939-s001.zip › ijms-1696530-supplementary.pdf]

Table S1. Body composition in female mice after 8 weeks on control diet (CD) or high fat/high sucrose (HF/HS) diet.

	**wt-CD (n=7)**	**APNhet-CD (n=8)**	**APNko-CD (n=9)**	**wt-HF/HS (n=9)**	**APNhet-HF/HS (n=8)**	**APNko-HF/HS (n=7)**	**two-way ANOVA**
Body weight (g)	18.9 ± 0.6	19.7 ± 0.7	20.8 ± 1.2	22.7 ± 0.9	24.4 ± 1.1	24.6 ± 1.3	a $F_{(1, 38)}$=20.36, *P*<0.0001
Fat mass (%)	13.9 ± 0.3	15.9 ± 0.2	16.2 ± 1.1	24.1 ± 1.7	23.7 ± 2.3	26.6 ± 2.2	a $F_{(1, 38)}$=43.45, *P*<0.0001
Lean mass (g)	16.3 ± 0.5	16.6 ± 0.6	17.4 ± 0.8	17.1 ± 0.4	18.5 ± 0.7	18.0 ± 0.7	
b-glucose (mmol/L)	6.4 ± 0.4	5.9± 0.3	6.8± 0.3	7.1 ± 0.4	7.1± 0.3	7.0± 0.3	a $F_{(1, 38)}$= 4.61, *P*=0.038
s-insulin (ug/L)	0.22 ± 0.04	0.43 ± 0.06*	0.45 ± 0.07 *	0.80± 0.20	0.45 ± 0.09	0.70 ± 0.13	a $F_{(1, 38)}$=6.65, *P*=0.014

The main effect of diet and genotype; adiponectin knockout (APNko), adiponectin heterozygote (APNhet), and wild-type (wt) was analyzed by two-way ANOVA. a; main effect of diet, there was no interaction effect. The effect of genotype within diet was analyzed using Brown-Forsythe ANOVA test with Dunnett´s multiple comparisons test. * P<0.05 vs wt within diet. Data are presented as mean ± SEM.

Table S2. RT-PCR primer sequences

Gene	Forward primer 5´-3´	Reverse primer 5´-3´
Actb	GACCCAGATCATGTTTGAGA	GAGCATAGCCCTCGTAGAT
Gapdh	ATGGCCTTCCGTGTTCCTAC	GCCTGCTTCACCACCTTCTT
Hprt1	CCCTGGTTAAGCAGTACAGCCCC	AGTCTGGCCTGTATCCAACACTTCG
Lpl	TGGATGAGCGACTCCTACTTCA	CGGATCCTCTCGATGACGAA
Cd36	GGTCCTTACACTACAGAGTTCGTTA	CATTGGGCTGTACAAAAGACACA
Slc7a5	GGCGTACATCAGCGTCAT	GTTCACGTCCTCAAGACTGTT
Slc38a4	TCACACTGCTGTTTCCAAGG	CAGCCGGAAGAATGAAAATC
Snat2	GCAGCCGGAGAAGGATGATGAAC	GAAGAGGGCGGCAAGCAAATACA
Slc2a3	ATGGGGACAACGAAGGTGAC	GTCTCAGGTGCATTGATGACTC
Pparγ	TTGAGTGCCGAGTCTGTGG	GGCATTGTGAGACATCCCCA
Ppargc1α	TATGGAGTGACATAGAGTGTGCT	CCACTTCAATCCACCCAGAAAG
Vegf	ATGCTCCTCCTAGTAACTGTGTCTGACT	CTCGAAGCCACAGGCTGATG
Prokr1	GCCATTGCCATTGACAGGTA	TGGTGAAGTAGGCAGCTGGA

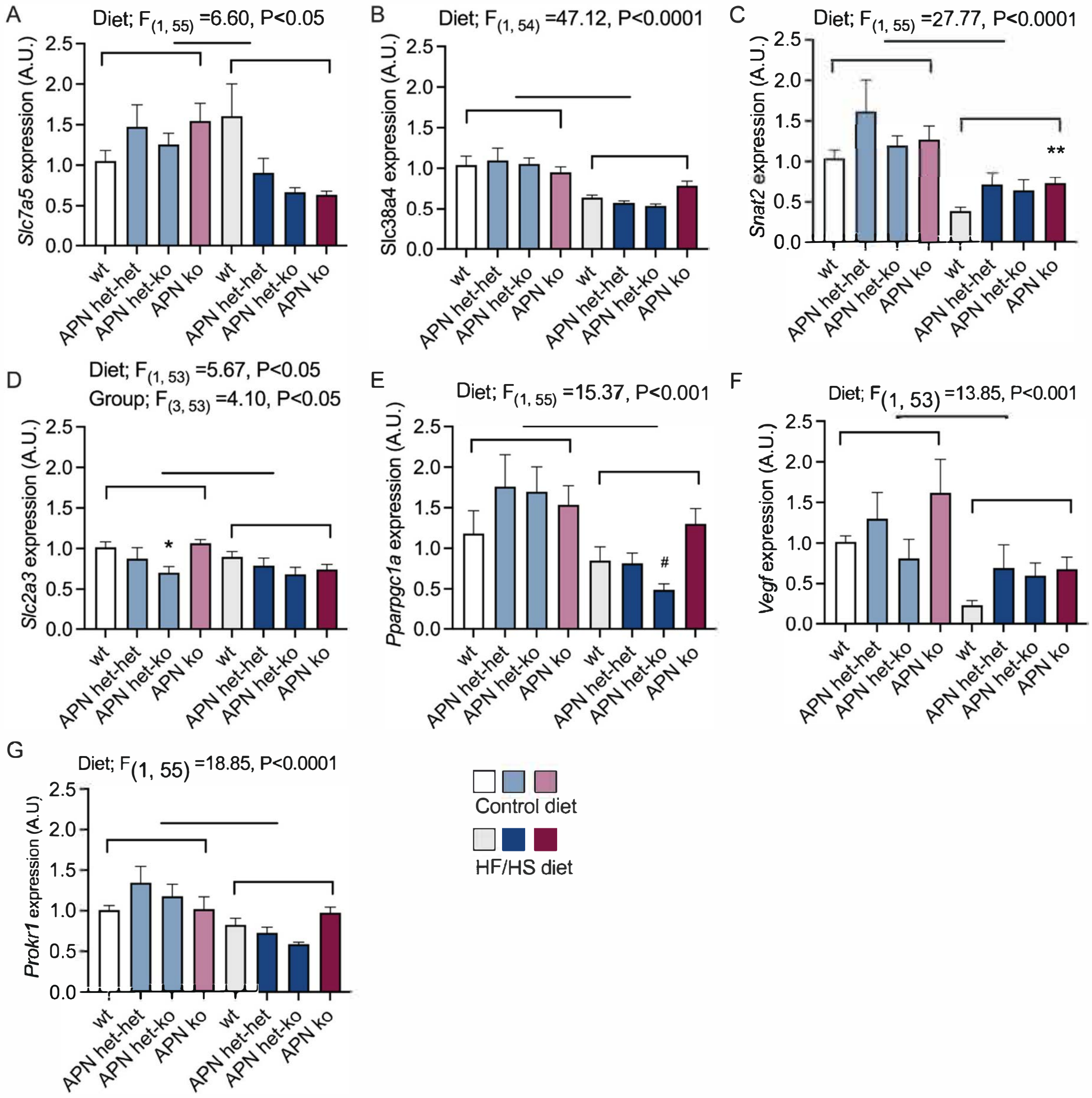

Supplemental Figure S1. Placenta gene expression of *Slc7a5* (A), *Slc38a4* (B), *Snat2* (C), *Slc2a3* (D), *Pparpgc1a* (E), *Vegf* ((F), and *Prokr1* (G) from adiponectin knockout (APN ko), adiponectin heterozygote (APN het), and wild-type (wt) dams on control or high-fat/high-sucrose (HF/HS) diet. The effect of diet-induced obesity and dam genotype (wt, APN het, and APN ko) was analyzed using two-way ANOVA. The effect of genotype within diet was analyzed using Brown-Forsythe ANOVA test with Dunnett´s multiple comparisons test. ** P<0.01 vs wt within diet. #P<0.05 APN het-het vs APN het-ko. Data are presented as mean ± SEM.
